# Supplementary material for: Synthesis of Well-Defined Gold Nanoparticles Using Pluronic: The Role of Radicals and Surfactants in Nanoparticles Formation
Source: Polymers (Basel). 2019 Sep 24;11(10):1553. doi: 10.3390/polym11101553 (PMC6835800; doi:10.3390/polym11101553)
Supplement: Supplementary file 1 [file polymers-11-01553-s001.pdf]

Supporting <u>information</u>

Synthesis of Well-Defined Gold Nanoparticles Using Pluronic: The Role of Environmental Parameters, Reactants and Reaction Products

M. Sokolsky-Papkov[1] and A.V. Kabanov[1,2,*]

1. Division of Molecular Pharmaceutics, Eshelman School of Pharmacy, University of North Carolina at Chapel Hill, Chapel Hill, NC 27599-7362, United States
2. Laboratory of Chemical Design of Bionanomaterials, Faculty of Chemistry, M.V. Lomonosov Moscow State University, Moscow 119992, Russia

Table S1: Characteristics of Pluronic block copolymers used in the experiment.

Copolymer	Mw	Average no. of PO units[1]	Average no. of EO units[1]	HLB[2]	CMC, M[3]
F127	12,600	65.2	200.4	22	$2.8*10^{-6}$
P104	5,900	61.0	53.6	13	$3.4*10^{-6}$
P85	4,600	39.7	52.3	16	$6.5*10^{-5}$
F88	11,400	39.3	207.8	28	$2.5*10^{-4}$

[1] The average numbers of EO and PO units were calculated using the average molecular weights (M_W) provided by the manufacturer.
[2] HLB of the copolymers were determined by the manufacturer.
[3] CMC values are taken from previously reported data[1].

Table S2: Physicochemical characteristics and ROS levels in F127 aqueous solutions[1]

Batch	F127 solution before lyophilization						Lyophilized and restored F127 solution					
	pH	D_{eff} (nm)[2]	PDI[2]	Free radicals (*10^3)[3]	Hydro-peroxides (*10^3)[3]	Hydro-peroxides/ Free radicals	pH	D_{eff} (nm)[2]	PDI[2]	Free radicals (*10^3)[c]	Hydro-peroxides (*10^3)[3]	Hydro-peroxides/ Free radicals
F127_1	6.9	28 ± 2	0.36 ± 0.05	15.6	33.96	2.17	6.9	28 ± 3	0.12 ± 0.02	18.9	38.4	2.03
F127_2	6.8	27 ± 3	0.58 ± 0.02	21.8	15.5	0.71	6.7	44 ± 3	0.16 ± 0.004	19.7	30.9	1.57
F127_3	5.1	62 ± 7	0.17 ± 0.03	21.9	60.1	2.74	6.7	35 ± 6	0.17 ± 0.01	18.1	11.6	0.64
F127_3	6.5[4]	74 ± 11	0.23 ± 0.05	21.9	40.31	1.84	n/a	n/a	n/a	n/a	n/a	n/a
F127_3	11.6[4]	81 ± 14	0.28 ± 0.08	26.4	64.01	2.43	n/a	n/a	n/a	n/a	n/a	n/a

[1] The 12.6% stock solutions of F127 solutions were prepared by dissolving the copolymer in bidistilled water. These solutions were lyophilized and restored to the same volume by bidistilled water.
[2] Micelles size and PDI were measured by DLS.
[3] The free radical and hydroperoxide levels were measured by EPR after mixing the same aqueous solutions of the copolymer with the equal volume of the bidistilled water (final concentration of the copolymer was 6.3%).
[4] The pH was adjusted with 1N NaOH.

Table S3: Characteristics of the unpurified GNP formed using non-lyophilized and lyophilized F127 samples[1].

Batch	Reaction mixture of native solutions			Reaction mixture of lyophilized and restored solution	
	D_{eff} (nm) [2]	PDI [2]	ζ-potential (mV) [2]	D_{eff} (nm) [1]	PDI[1]
F127_1	254 ± 3	0.235±0.01	-6.38±0.16	105 ± 1	0.485±0.016
F127_2	460 ± 5	0.07±0.03	-2.1±0.18	148 ± 2	0.17±0.006
F127_3	466 ± 9	0.46±0.12	-1.51±0.16	102 ± 1	0.506±0.007
F127_3 (pH 6.5)	283 ± 5	0.215±0.01	-4.99±0.38	n/a	n/a
F127_3 (pH 11)	44 ± 1	0.611±0.003	-7.63±0.47	n/a	n/a

[1] The 12.6% stock solutions of F127 solutions were prepared by dissolving the copolymer in bidistilled water. These solutions were lyophilized and restored to the same volume by bidistilled water. GNP were synthesized by mixing equal volumes of non-lyophilized and lyophilized F127 solutions and $HAuCl_4$ solution (final concentrations 6.3% w/w F127 and 0.25 mM $HAuCl_4$) followed by incubation of the mixture for 2h at 45°C.

[2] Particle size, PDI and ζ-potential were measured by DLS.

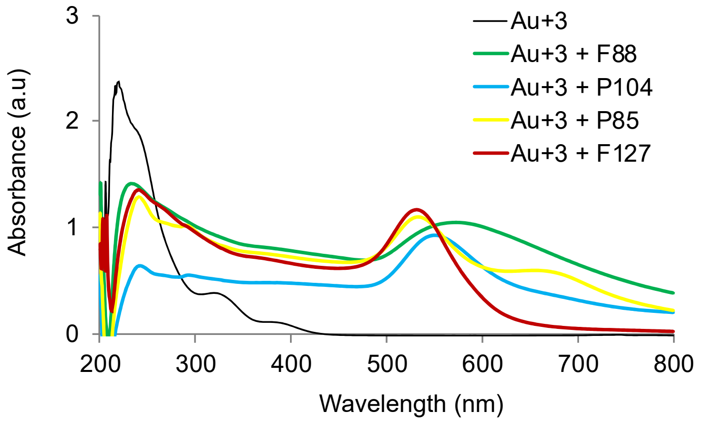

Figure S1: Absorbance spectra recorded in the initial $HAuCl_4$ aqueous mixtures containing 5 mM Pluronic P104, F127, F88 and P85 and 0.25 mM $HAuCl_4$ 2 h after incubation at 25°C. The copolymer and $HAuCl_4$ concentrations were kept constant with the copolymer concentration being well above its CMC and at the 20-fold molar excess compared to $HAuCl_4$.

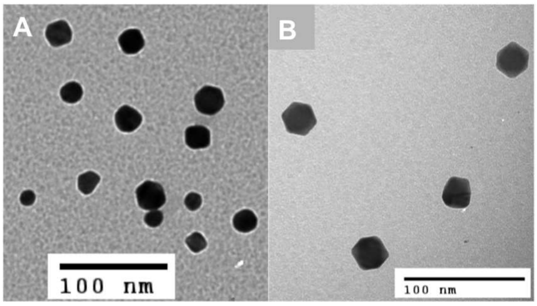

Figure S2: TEM images of the purified gold nanoparticles (GNP) synthesized at **(A)** 25°C or **(B)** 45°C. Particles were synthesized by mixing equal volumes of F127 and $HAuCl_4$ solutions (final concentrations 6.3% w/w and 0.25 mM respectively) followed by incubation of the mixture for 2h at 25° or 45°C. Reaction mixture was purified from excess of the polymer as described in **Table 1.**

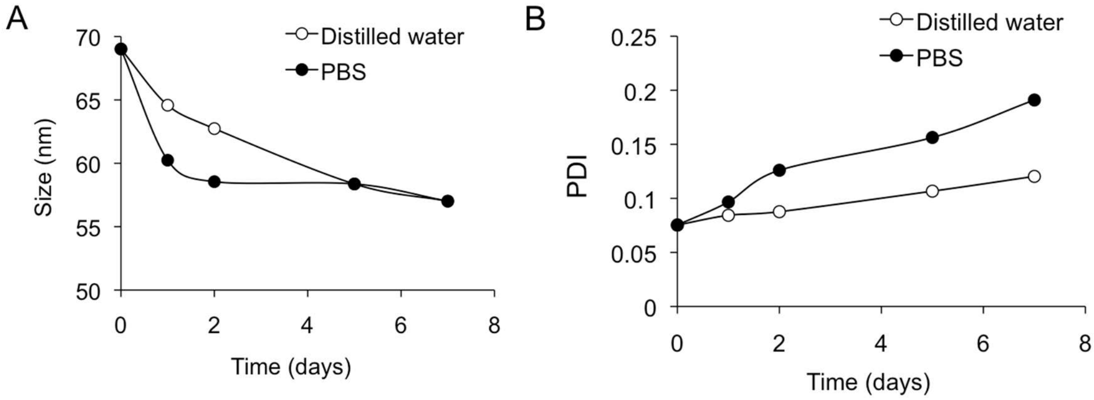

Figure S3: Stability of purified GNP in double distilled water (DDW) or PBS. Particles were synthesized by mixing equal volumes of F127 and $HAuCl_4$ solutions (final concentrations 6.3% w/w and 0.25 mM respectively) followed by incubation of the mixture for 2h at 45°C. Reaction mixture was purified from excess of the polymer as described in **Table 1** and restored with DDW or PBS respectively. The nanoparticle concentration remained the same before and after purification.

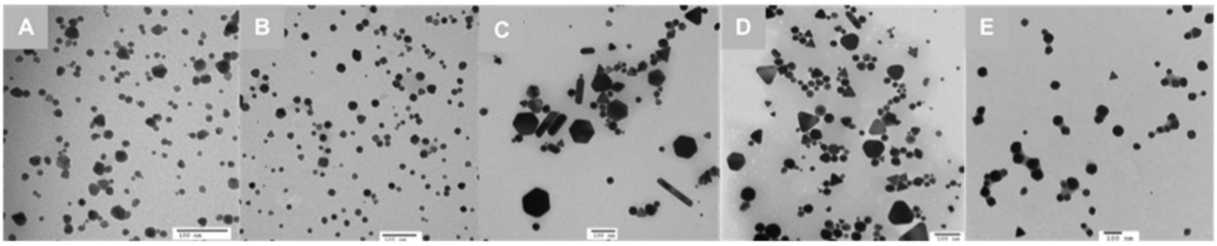

Figure S4: TEM images of the purified GNP synthesized at 45°C in the presence of 6.3% F127 at various concentrations of $HAuCl_4$: **(A)** 0.05 mM, **(B)** 0.1 mM, **(C)** 0.15 mM, (D) 0.2 mM, **(E)** 0.25 mM. All TEM bars are 100 nm.

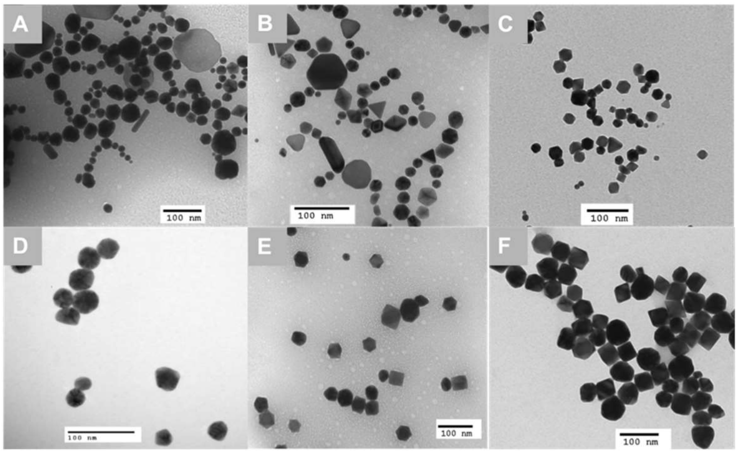

Figure S5: TEM images of the purified GNP synthesized at 45°C in 0.25 mM $HAuCl_4$ solution in the presence of various concentrations of F127: **(A)** 1%, **(B)** 2%, **(C)** 5%, **(D)** 6.3%, **(E)** 7.5%, and **(F)** 10% w/w. All TEM bars are 100 nm.

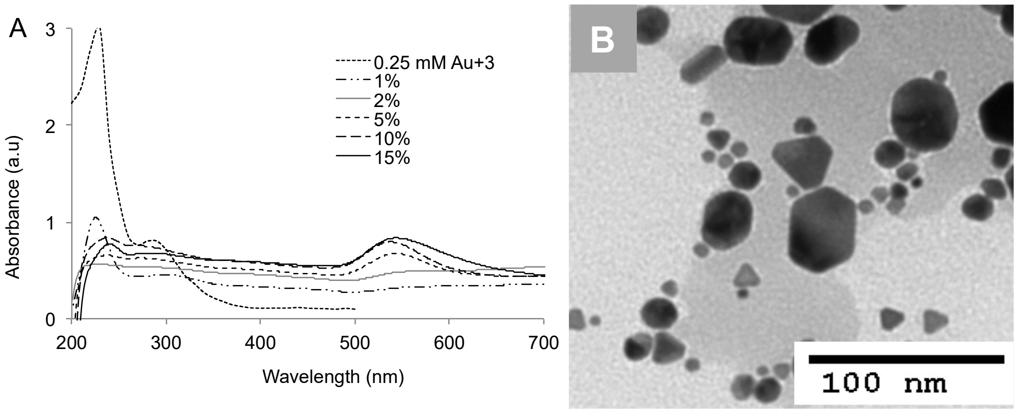

Figure S6: (A) Absorbance spectra of the reaction mixtures of 0.25 mM $HAuCl_4$, and various concentrations of PEO 8000 6 h after incubation at 45°C; **(B)** TEM images of the purified GNP formed at 10% w/w PEO 8000.

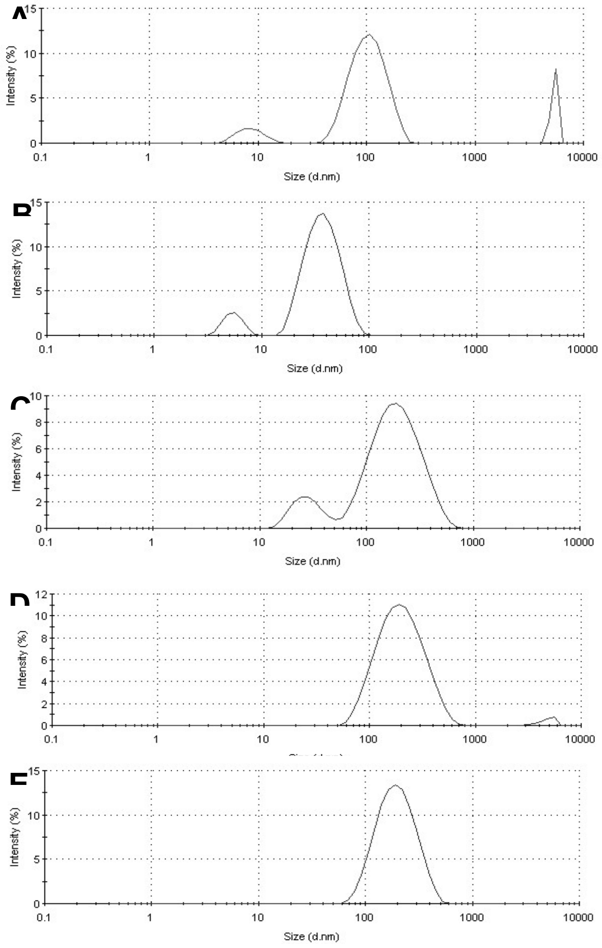

Figure S7: DLS analysis of the mixtures containing 6.3% w/w F127 and 0.25mM of $HAuCl_4$ at 25°C: **(A)** 0.25mM $HAuCl_4$ **(B)** 6.3% w/w F127 **(C)** 20 min, **(D)** 60 min, **(E)** 120 min

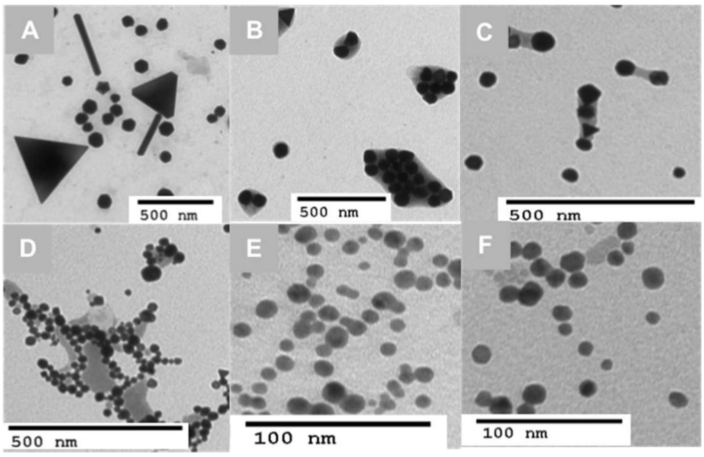

Figure S8: EM images of the purified GNP synthesized in the presence of 6.3% w/w F127 and 0.25 mM $HAuCl_4$ at different pH: **(A)** pH 2.2, **(B)** pH 4.5, **(C)** pH 6.7, **(D)** pH 7.5, **(E)** pH 9.0, and **(F)** pH 11.5.

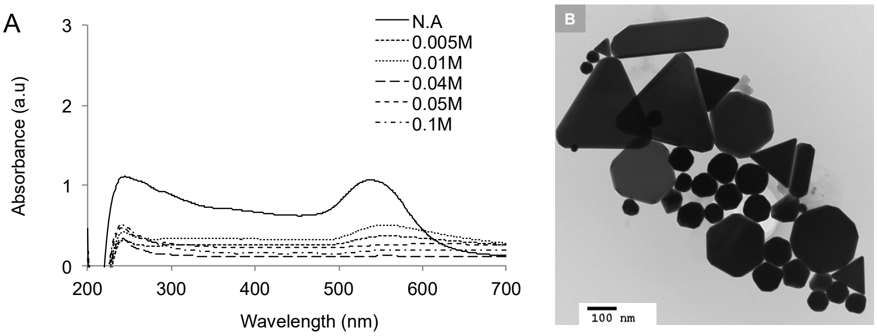

Figure S9: (A) Absorbance spectra of the reaction mixtures of 6.3% F127 and 0.25 mM $HAuCl_4$, 6.3% with various concentrations of NaCl 6 h after incubation at 45°C; **(B)** TEM image of the purified GNP synthesized in the solutions of 6.3% w/w F127 and 0.25 mM $HAuCl_4$ in the presence of 0.005 M NaCl at 45°C. Reaction mixture was purified from excess of the polymer as described in **Table 1**.

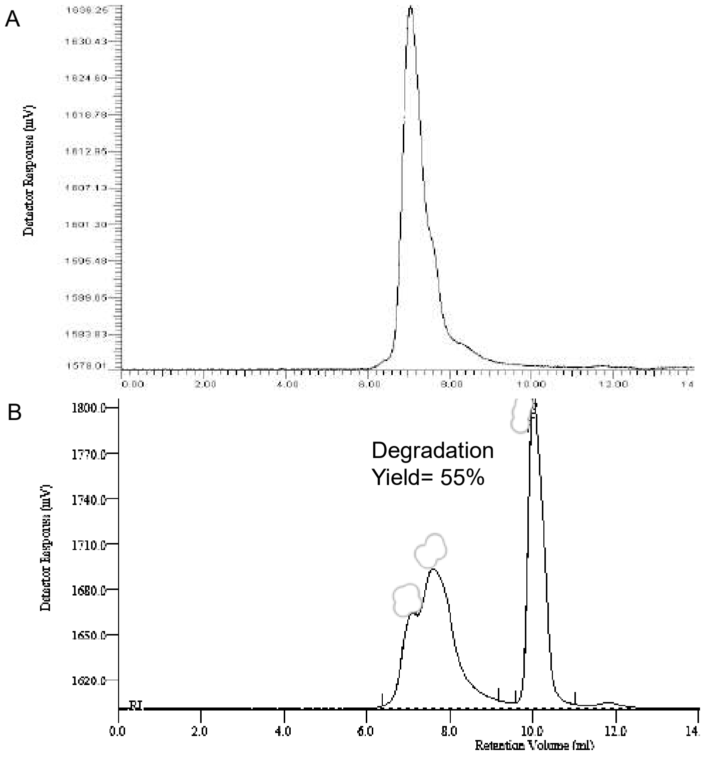

Figure S10: Gel permeation chromatography of **(A)** original F127 (0.5 mM) and **(B)** separated reaction products. Mobile phase: 0.02% sodium azide in distilled water Viscotek G3000PWXL column.

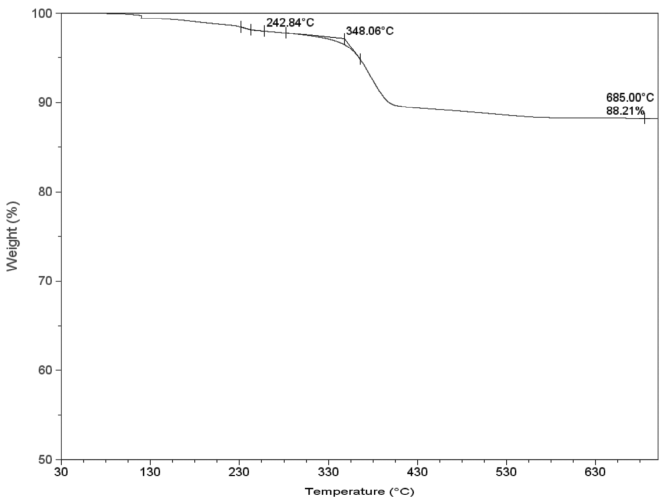

Figure S11: TGA of purified GNP. Particles were synthesized by mixing equal volumes of F127 and $HAuCl_4$ solutions (final concentrations 6.3% w/w and 0.25 mM respectively) followed by incubation of the mixture for 2h at 45°C. Reaction mixture was purified from excess of the polymer as described in **Table 1** and the particles were lyophilized to dryness.

References:

1. Batrakova, E. V.; Li, S.; Alakhov, V. Y.; Miller, D. W.; Kabanov, A. V., Optimal structure requirements for pluronic block copolymers in modifying P-glycoprotein drug efflux transporter activity in bovine brain microvessel endothelial cells. *J. Pharmacol. Exp.Ther.* **2003**, *304*, 845-854.
